# Evaluating Vaccination Status and Barriers in Children with Rheumatic Diseases

**DOI:** 10.3390/vaccines13040384

**Published:** 2025-04-03

**Authors:** Shine Vazhappilly, Babatope O. Adebiyi, Racheal Githumbi, Nicole A. Johnson, Otto G. Vanderkooi, Heinrike Schmeling

**Affiliations:** Department of Paediatrics, Cumming School of Medicine, University of Calgary, Calgary, AB T2N 1N4, Canada; shine.v@hotmail.com (S.V.); babatope.adebiyi@ucalgary.ca (B.O.A.); racheal.githumbi@ucalgary.ca (R.G.); nicole.johnson@albertahealthservices.ca (N.A.J.); otto.vanderkooi@albertahealthservices.ca (O.G.V.)

**Keywords:** pediatric rheumatic diseases, immunization barriers, pediatric vaccination

## Abstract

**Background**: This study aims to evaluate the vaccination status of children with rheumatic diseases (RD) compared to healthy controls (HC) and immunization barriers, as studies examining the vaccination status and factors promoting or hindering vaccination among children RD remain limited. **Methods**: A cross-sectional study was conducted on children with RD (in a rheumatology clinic) and HC (in a fracture clinic) at a tertiary care center in Canada. Demographics, diagnosis, treatments, and vaccine status were obtained from health records and a provincial electronic vaccine database. A patient/caregiver questionnaire was used to capture perceived immunization barriers, concerns, and satisfaction. Descriptive statistical methods were used for analysis. **Results**: The study involved 144 children with RD and 111 HC. Data from 94 children with RD and 86 HC, all lifelong Alberta residents, were analyzed for objective vaccination status. Most vaccines were received at rates of 80% or higher, except the influenza vaccine, which had the lowest adherence (34% in RD vs. 21% in HC). In 31% of RD children, vaccinations were withheld due to active disease, healthcare provider advice, or caregiver concerns about side effects. In 27% HC, vaccinations were withheld due to side effects. Both groups primarily relied on their family doctor for vaccination information, and 85% or more expressed satisfaction with the information received. **Conclusions**: Most children with RD and HC received recommended vaccines, but influenza vaccination gaps were identified. Knowledge about vaccine contraindications in RD is well understood, but perceived safety concerns limit vaccination completeness. Healthcare providers, especially family doctors, pediatricians, and rheumatologists, should be providing education resources for vaccines and be proactive in discussing the safety and necessity of vaccinations.

## 1. Introduction

Childhood rheumatic diseases are a diverse group of autoimmune inflammatory disorders thought to be a result of complex interactions between genetics and the environment. Patients with rheumatic diseases have an increased incidence of infections and their complications, both from underlying disease activity and immunosuppressive treatments [1]. Vaccines have been well-established as effective intervention in combating these infections.

Published International and National guidelines for immunocompromised pediatric patients differ slightly from vaccination schedules published for the healthy pediatric population. The European League Against Rheumatism (EULAR) guidelines for immunization in pediatric rheumatic diseases [2,3] suggests that inactivated vaccines are efficacious and well tolerated. Also, live attenuated vaccines may be less effective and may potentially exacerbate the underlying illness [4], though recent recommendations suggest these vaccines can be administered after an appropriate washout period from immunosuppressive medications [3].

Vaccination coverage in pediatric rheumatic diseases (PRD), suggests that compliance is variable. Past and more recent Juvenile Idiopathic Arthritis (JIA) cohorts’ studies maintain that vaccine completeness is suboptimal [5,6,7]. This finding persists in multiple global contexts [8,9,10,11] where adolescent PRD patients consistently had lower proportions of vaccination completeness. These differences were exacerbated in studies that compared pediatric rheumatic diseases (PRD) with healthy controls and/or patients not on immunosuppressive therapies [6,11].

Studies assessing vaccination barriers in pediatric immune-mediated diseases remain limited and non-specific to PRD. Barriers, more extensively evaluated in adult populations, cite vaccine hesitancy in the adult rheumatic to primarily result from a fear of adverse reactions and a lack of recommendation from the treating physician [4]. Other adult studies highlighted that concerns regarding vaccine effectiveness and safety were higher in rheumatic disease patients who had not received many recommended vaccines [12,13].

As such, this study aims to address these gaps by comparing the vaccination status between pediatric rheumatic disease (RD) patients and healthy patients (HC), as well as to identify current immunization knowledge and barriers faced by both populations.

## 2. Methods

### 2.1. Study Design

This cross-sectional study evaluated the vaccination status and barriers among children with rheumatic diseases attending a single tertiary care rheumatology clinic in Alberta, Canada.

### 2.2. Population

Patients <18 years with confirmed rheumatic diseases were randomly approached at regular rheumatology clinic visits and asked for voluntary participation. The healthy control group consisted of children (<18 years) who were followed at the Orthopedics Fracture clinic and did not have a RD.

### 2.3. Data Collection

Demographic Variables: sex, age, diagnoses, and current immunosuppressive therapies were obtained from health records.

Vaccination Status: The provincial electronic vaccine database—a system used to track and manage vaccination records that are administered in Alberta only—was used to obtain objective participants vaccination history.

Immunization Barriers and Sources: A survey (patient/parent questionnaire) distributed during regular clinic visits was used to assess perceived immunization barriers, concerns, knowledge regarding contraindications to vaccination, sources of immunization information, and satisfaction with information received. This survey was developed, edited, and finalized by following the completion of an in-depth literature review and iterative feedback rounds from various rheumatologists, infectious disease specialists, rheumatology nurses, parents, and patients. The process above ensured validity of our survey. Also, we asked some of the parent/caregivers to answer the questionnaire for a second time. Afterward, we compared their responses to show consistency. Furthermore, we calculated Cronbach’s alpha for the study. The Cronbach’s alpha was 0.9.

### 2.4. Ethical Statement

This study has been approved by the Conjoint Health Research Ethics Board (REB13-1290) of the University of Calgary (Calgary, AB, Canada). Informed consent was obtained from all patients and/or their legal guardians.

### 2.5. Analysis

Data were analyzed using descriptive statistics (mean, median, interquartile range-Q25/Q75, proportions).

## 3. Results

### 3.1. Patient Characteristics

The study enrolled 255 participants, 144 with childhood rheumatic diseases and 111 healthy controls as presented in Table 1. Most of the patients with RD were diagnosed with juvenile idiopathic arthritis (79%). Other diagnoses included systemic lupus erythematosus, celiac disease, autoimmune encephalitis, and vasculitis.

### 3.2. Vaccination Status

Vaccination status was evaluated based on the age-specific guidelines published by Alberta Health Services [14]. At the time of the study, the guidelines recommend routine vaccinations from birth to 18 years to protect against infectious diseases. Infants receive vaccines for diphtheria, tetanus, pertussis, polio, Haemophilus influenzae type b, pneumococcus, meningococcus, rotavirus, measles, mumps, rubella, and varicella. At 4 years, a booster for tetanus, diphtheria, pertussis, and polio is given. In Grade 6 (11–12 years), hepatitis B and HPV (only recommended for girls) vaccines are administered, while Grade 9 (14–15 years) includes Tdap and meningococcal vaccines. Annual influenza vaccines are recommended for all ages. Also, adherence refers to the extent to which individuals follow the recommended vaccination schedule as outlined.

In Alberta, vaccinations are recommended but not mandatory. For the pre-school population, vaccines are administered at public health vaccine clinics, while for the school-age population, they are given at school. In Alberta, all vaccines are scheduled on a routine basis, except for influenza. However, influenza vaccines are available at public health clinics, pharmacies, and primary health providers.

To determine objective and accurate vaccination status, data from 94 of 144 patients (65%) with RD were analyzed, representing individuals born and living in Alberta their entire lives (based on vaccine registry). Adherence rates for most vaccines, except HPV and influenza, were near or above 80%, with influenza having the lowest rate at 34%. For HC, data from 86 of 111 (77%) participants were analyzed to represent individuals born and living in Alberta their entire lives. Most vaccines had adherence rates of 85% or higher, except for the influenza vaccine, which had the lowest rate at 21%.

Interestingly, the adherence of HPV in the disease group was lower than in the control group (69% vs. 89%) (Table 2).

**Table 2:** Specific vaccination adherence in disease and control groups according to Alberta’s vaccine registry.

**Rheumatic Disease Group**				
**Vaccine Type**	**0–4 Years Old (%)** **n = 8**	**5–11 Years Old (%)** **n = 38**	**12+ Years Old (%)** **n = 48**	**Adherence Rate %** **n = 94**
Polio	8 (100%)	28 (74%)	43 (90%)	79 (84%)
DTaP	8 (100%)	28 (74%)	42 (88%)	78 (83%)
HiB	8 (100%)	35 (92%)	43 (90%)	86 (91%)
MMR	8 (100%)	28 (74%)	47 (98%)	83 (88%)
Varicella	8 (100%)	34 (89%)	42 (88%)	84 (89%)
Pneumococcal	8 (100%)	35 (92%)	48 (100%)	91 (97%)
Meningococcal	8 (100%)	32 (84%)	34 (71%)	74 (79%)
Hepatitis B *	N/A	N/A	45 (94%)	45/48 (94%)
HPV *^	N/A	N/A	22/32 (69%)	22/32 (69%)
Influenza (annually)	2 (25%)	12 (32%)	18 (38%)	32 (34%)
**Healthy Controls**				
**Vaccine Type**	**0–4 Years Old (%)** **n = 14**	**5–11 Years Old (%)** **n = 33**	**12+ Years Old (%)** **n = 39**	**Adherence Rate %** **n = 86**
Polio	14 (100%)	30 (91%)	36 (92%)	80 (93%)
DTaP	14 (100%)	30 (91%)	36 (92%)	80 (93%)
HiB	14 (100%)	30 (91%)	35 (90%)	79 (92%)
MMR	14 (100%)	32 (97%)	38 (97%)	84 (98%)
Varicella	13 (93%)	32 (97%)	37 (94%)	82 (95%)
Pneumococcal	13 (93%)	27 (82%)	37 (94%)	77 (90%)
Meningococcal	13 (93%)	28 (85%)	32 (82%)	73 (85%)
Hepatitis B *	N/A	N/A	28/31 (90%)	28/31 (90%)
HPV *^	N/A	N/A	17/19 (89%)	17/19 (89%)
Influenza	6 (43%)	8 (24%)	4 (10%)	18 (21%)

* Based on age-specific guidelines. ^ Only girls were recommended to receive this vaccine.

### 3.3. Survey Evaluation

All participants who were enrolled in the study successfully completed the questionnaire, and no subjects were excluded due to missing or incomplete data. In the survey, 44/144 (31%) patients with RD and 30/111 (27%) healthy controls (HC) reported missing at least one vaccination (Table 3). In addition to reasons in Table 3, other reasons cited by patients included beliefs about the influenza vaccine ineffectiveness, concerns about future disease, uncertainty, vaccine unavailability, personal reasons (HPV), existing illness (Varicella), and active infections requiring antibiotics (missed the influenza vaccine). Among the controls, reasons included media influence, perception of too many vaccines, influenza vaccine ineffectiveness, and insufficiently compelling studies.

### 3.4. Information Sources and Satisfaction

The study found that patients and families primarily sourced vaccination information from family doctors as presented in Table 4. At least 85% of respondents in both groups received enough information about immunizations, with 50% being very satisfied. Less than 5% were somewhat or very dissatisfied with the information they received.

## 4. Discussion

This is the first study to our knowledge evaluates both the objective vaccination status (vaccine registry) and immunization barriers in a pediatric rheumatic cohort, comparing these findings to a healthy control group. Notably, most vaccinations in both groups, apart from influenza, were received at rates near or above 80–85%.

The rate of overall vaccination completeness was slightly lower in the RD group. Interestingly, the adherence of HPV in the disease group was lower than in the control group (69% vs. 89%).

The primary reason for missed vaccinations in the RD group was concern for active disease, while in the healthy control (HC) group, it was related to fears of vaccination side effects. Despite these barriers, 85% of respondents in each group expressed satisfaction with the amount and quality of information they received about vaccinations.

The vaccination rates for patients with RD in this study are higher compared to those reported in similar studies. For instance, a study in Russia on children with juvenile idiopathic arthritis (JIA) reported vaccination completeness rates between 40% and 75.8% [6]. A longitudinal, observational multicenter cohort study in Switzerland found an overall vaccination completeness of only 3.8% among pediatric rheumatic disease participants [11]. In Quebec, a study on JIA patients showed a 61% vaccination status by their last clinic visit, though this excluded varicella, HPV, and influenza vaccines [7]. Similarly, a Slovenian study reported a 65% complete vaccination status in their rheumatic disease group [9], while a Polish study found a 49% complete vaccination rate among JIA patients [15].

Comparing these studies is challenging due to differences in inclusion criteria, vaccination evaluation methods, and the types of vaccines assessed. Variations in patient populations, sample sizes, and study designs also affect the results. Factors such as geographical location, healthcare system differences, and access to vaccines can influence findings. The period of the study and the definition of vaccination completeness (e.g., national guidelines vs. special recommendations for immunocompromised patients) also vary. Data sources like medical records, self-reports, or vaccination registries can introduce biases. Additionally, the focus of each study (e.g., specific vaccines or broader vaccination schedules) and the treatment status of patients, particularly immunosuppressive therapy, can impact outcomes. Sociocultural factors, such as parental attitudes and healthcare provider recommendations, further contribute to variability.

In addition to these methodological differences, Alberta’s publicly funded immunization program and strong healthcare infrastructure may contribute to the higher vaccination rates observed in our study. Routine childhood vaccinations are administered in public health vaccine clinics for pre-school age children and formed part of school-based programs for school-age children, ensuring accessibility and timely administration. Additionally, the use of an electronic vaccine registry provides a reliable and standardized method for tracking immunization status, reducing recall bias that may affect studies relying on self-reported data. Differences in healthcare policies, vaccine funding, and accessibility across countries could further explain the observed variations. Future research should explore how these systemic factors influence vaccine adherence to inform strategies for improving immunization rates globally.

The most missed vaccination in our RD and HC groups is influenza, despite it being recommended for both groups in our province. In another rheumatic disease cohort, the adherence rate was even lower with only 10.2% [9]. But notably, the influenza vaccination is an annual vaccination, and adherence with this vaccine might be challenging, and subjects might receive one less than once per year. Additionally, with the HPV vaccine, we observed a lower adherence rate in the RD group compared to the HC group. The fact that the HPV vaccine is administered at an age (12+ years) when many in the RD group had already been diagnosed could have increased the fear of a potential flare of their disease. However, we cannot categorically state that this is the reason. Therefore, in future studies the reasons for these results can be explored in larger populations.

In our study, barriers to vaccination differed between the RD and HC groups. The RD group was primarily concerned about active disease, while the HC group focused on vaccination safety. Findings from many studies align with our study. Lawson et al. [16] demonstrated that errors of omission and failure to address patient concerns were common reasons for missed vaccinations in SLE patients. In China, vaccination adherence among rheumatic disease patients is very low, with barriers including perceived lack of benefit, incorrect beliefs about the seriousness of infections, lack of doctor recommendations, and safety concerns [13]. In Mexico, patients reported barriers such as not receiving a recommendation from their rheumatologist, finding the vaccine unavailable, believing vaccines are ineffective, fearing adverse events, having previously experienced adverse events, and other reasons [12].

### 4.1. Recommendations

Healthcare providers, especially family doctors and pediatricians, should be providing education resources for vaccines and should be proactive in discussing the safety and necessity of vaccinations, including during periods of active rheumatic diseases.

Additionally, improving access to vaccinations through convenient scheduling and follow-up reminders can help increase adherence rates, such as by utilizing a dedicated vaccination center. In such centers, individuals would receive accurate information about vaccine safety and current guidelines. These centers would also offer the opportunity to accurately document vaccines and potential side effects, a benefit provided by the vaccine registry in our province. Furthermore, reinforcing the role of trusted medical professionals as primary sources of immunization information and ensuring the availability of comprehensive, up-to-date vaccine information can support parents in making informed decisions. However, such a database is lacking in many provinces and countries.

### 4.2. Limitations of Study

This study has several limitations. First, participation was voluntary, which may induce selection bias due to overrepresentation of proactive individuals. To mitigate this, we used diverse recruitment methods to reach a broader range of participants. Recall bias may also affect the validity of the findings, as participants might not accurately remember past events. We reduced this by using validated questionnaires and cross-referencing data with available records where possible. The exclusion of children born outside of Alberta may limit the generalizability of the findings to immigrant populations; future studies should include a more diverse cohort. Although the sample size is sufficient for group comparisons, the study was confined to a single tertiary care center. Expanding to multiple centers in future research could improve generalizability. Finally, the cross-sectional design limits the ability to assess long-term vaccination adherence, suggesting the need for longitudinal studies in the future.

## 5. Conclusions

Our research reveals that most children with RD and HC have received their recommended vaccinations, although notable deficiencies exist, especially regarding influenza. Despite a solid understanding of contraindications, safety concerns, particularly amidst active disease, hinder vaccination rates in RD patients. There is a necessity for customized strategies to address these apprehensions and enhance adherence to influenza vaccinations, such as proactive dialogues between healthcare professionals and families during standard consultations. The strategies can also include focused education and regular follow-up to maximize vaccination uptake in at-risk pediatric demographics.

## Figures and Tables

**Table 1:** Patient and control characteristics.

		RD Number (%)	HC Number (%)
Patients		144	111
Median Age (Q25/Q75)		11 (8/14)	10 (6/14)
Females		105 (73)	46 (41)
Have a Family Doctor		137 (95)	80 (72)
Have a Pediatrician		61 (42)	16 (14)
Taking immune modifying drugs		109 (76)	N/A
	DMARDS *	97 (67)	N/A
	Biologics **	31 (22)	N/A
	Steroids ***	22 (15)	N/A

RD = Rheumatic Disease. HC = Healthy Controls. * Disease-modifying anti-rheumatic drugs (DMARDs) such as azathioprine, cyclophosphamide, cyclosporine, hydroxychloroquine, methotrexate, mycophenolate mofetil, leflunomide, and sulfasalazine. ** Such as adalimumab, certolizumab, etanercept, and tocilizumab. *** Such as prednisone and methylprednisolone.

**Table 3:** Reasons for missing vaccination.

Reason *	RD (n = 44)	HC (n = 30)
My child had an active disease.	12 (27%)	1 (3%)
I am concerned that vaccination can cause a disease flare.	6 (14%)	2 (7%)
I have concerns related to side effects after vaccine administration.	8 (18%)	4 (13%)
My child has experiences with negative reactions to vaccination in the past.	5 (11%)	2 (6%)
My child is not recommended to have vaccinations.	11 (25%)	0 (0%)
I am not sure if my child is allowed to receive certain vaccinations.	5 (11%)	1 (3%)
I did not know about the vaccine.	1 (2%)	1 (3%)
I did not remember/I missed it.	0 (0%)	0 (0%)
I was not told to have my child receive the vaccine.	1 (2%)	1 (3%)
Other	7 (16%)	7 (23%)

* Survey respondents had the option to select more than one answer.

**Table 4:** Sources of vaccine information *.

	RD (n = 144)	HC (n = 111)
Family Doctor or Pediatrician	94 (66%)	77 (69%)
Rheumatology Clinic	74 (52%)	0 (0%)
Nurses	51 (36%)	29 (26%)
Vaccination/Travel Clinic	51 (36%)	56 (50%)
Other Health Professionals	20 (14%)	5 (5%)
Internet	22 (15%)	39 (35%)
School	55 (38%)	50 (45%)
Family/Friends	27 (19%)	29 (26%)
Other	10 (7%)	5 (5%)

* Survey respondents could select more than one answer.

## Data Availability

The data are available within the manuscript.
